# Supplementary material for: Multivariate genome-wide association study on tissue-sensitive diffusion metrics highlights pathways that shape the human brain
Source: Nat Commun. 2022 May 3;13:2423. doi: 10.1038/s41467-022-30110-3 (PMC9065144; doi:10.1038/s41467-022-30110-3)
Supplement: Supplementary file 1 — Supplementary Information [file 41467_2022_30110_MOESM1_ESM.pdf]

Supplementary Information

Multivariate genome-wide association study on tissue-sensitive diffusion metrics highlights pathways that shape the human brain

Authors

Chun Chieh Fan[1,2,3*], Robert Loughnan[4], Carolina Makowski[2,3], Diliana Pecheva[2,3], Chi-Hua Chen[3], Donald J. Hagler, Jr.[2,3], Wesley K. Thompson[1,3], Nadine Parker[5], Dennis van der Meer[5,6], Oleksandr Frei[5,7], Ole A. Andreassen[5], Anders M. Dale[2,3,4,8]

1. Population Neuroscience and Genetics Lab, University of California San Diego, USA.

2. Center for Multimodal Imaging and Genetics, University of California San Diego, USA

3. Department of Radiology, School of Medicine, University of California San Diego, USA

4. Department of Cognitive Science, University of California San Diego, USA

5. NORMENT Centre, Division of Mental Health and Addiction, Oslo University Hospital & Institute of Clinical Medicine, University of Oslo, Oslo, Norway

6. School of Mental Health and Neuroscience, Faculty of Health, Medicine and Life Sciences, Maastricht University, The Netherlands

7. Centre for Bioinformatics, Department of Informatics, University of Oslo, Oslo, Norway

8. Department of Neuroscience, University of California San Diego, 9500 Gilman Drive, La Jolla, CA 92037, USA

Supplementary Discussion

Validated loci as druggable targets

To investigate how druggable our validated loci are, we queried the DGIdb [45] with all genes that mapped onto the validated loci (Supplementary Data 9-11). Among the validated loci, 218 of the N0 loci (64%) are druggable with 207 of them have known pharmacological interactions. This high degree of druggable targets was also 2observed among ND loci (185 loci are druggable and 175 of them have known interactions) and NF loci (125 loci are druggable and 125 of them have known interactions). The gene set analyses show those druggable genes were highly enriched in brain tissues, especially hippocampus (P_{bon} = 8.0e-15, 1.5e-13, 2.5e-7 for N0, ND, and NF, respectively) and amygdala (P_{bon}= 1.4e-13, 6.2e-15, 2.5e-19 for N0, ND, and NF, respectively). Those druggable genes were evidently overlaps with genes identified in neuropsychiatric disorders and immune disorders, such as schizophrenia (P_{bon}= 1.4e-8, 2.8e-9, 3.4e-6 for N0, ND, and NF), bipolar disorders (P_{bon}= 9.3e-9, 4.3e-9, 1.2e-4 for N0, ND, and NF), and inflammatory bowel disease (P_{bon}= 9.3e-14, 6.6e-14, 4.0e-12 for N0, ND, and NF).

Average heritability across three RSI metrics

Although each phenotypic PC can have heritabilities as high as 0.32 to 0.36 (Supplementary Figure 11), the average heritability of the combined PC for each imaging feature is modest. As the optimal heritability measures for high-dimensional multivariate measurements would be the mean heritability across PCs (See Method), we found that the mean signal for N0, ND, and NF are 0.09 (95%CI 0.04 - 0.13), 0.06 (95%CI 0.02 - 0.10), and 0.05 (95%CI 0.01 - 0.09). Given the sum of the variance explained by the independent loci found to be significant, the discovered loci reached 59%, 60%, and 58% of the average SNP-heritabilities for N0, ND, and NF.

Supplementary Table 1. Characteristics of discovery and validation samples

	UKB discovery (MRI before 2019)	UKB validation (MRI after 2019)	ABCD
N	23543	6396	8189
Age in years Mean(SD)	55.2 (7.3)	55.0 (7.3)	9.9 (0.6)
Sex (% of Males)	50%	43%	52%
Proportion of European ancestry	1	1	0.63
Proportion of Non-European ancestry			0.37

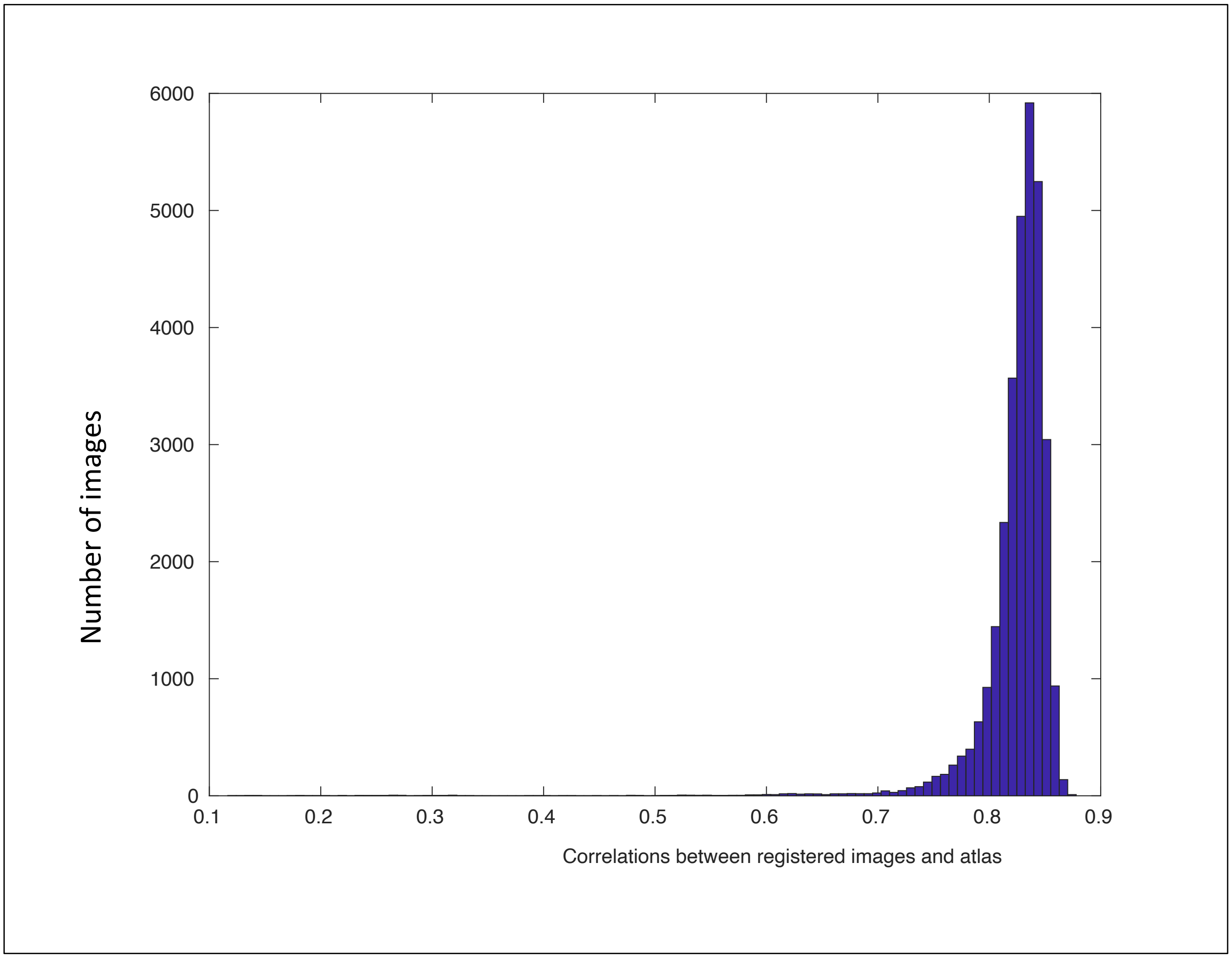

Supplementary Figure 1. Histogram of the registration quality metrics across all imaging samples.

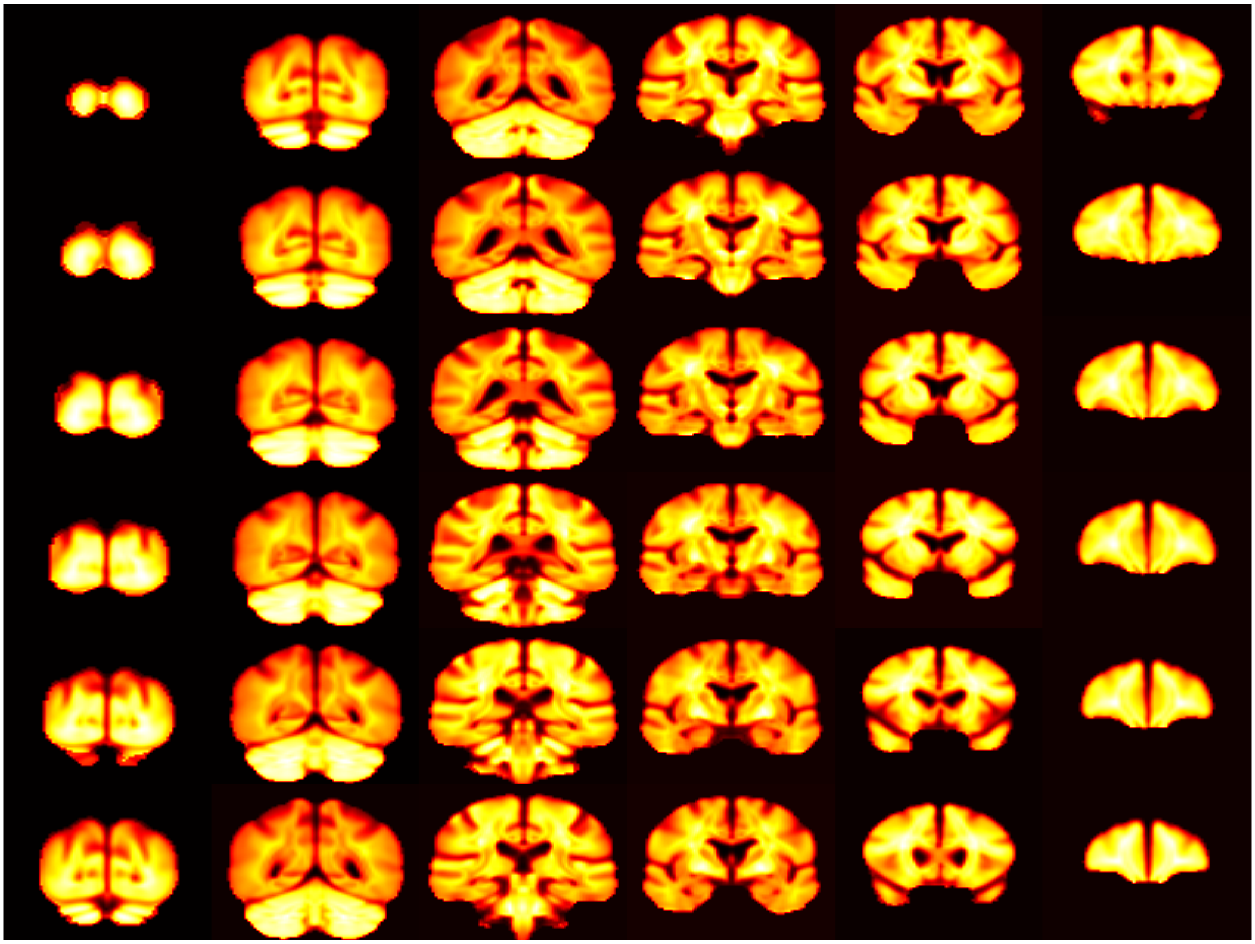

Supplementary Figure 2. Spatial distribution of N0. The N0 metrics were averaged across discovery samples of UKB. The coronal sections were rendered slice by slice for every 8 mm. Upper left, first slice from the posterior region. Lower left, last slice from the frontal region.

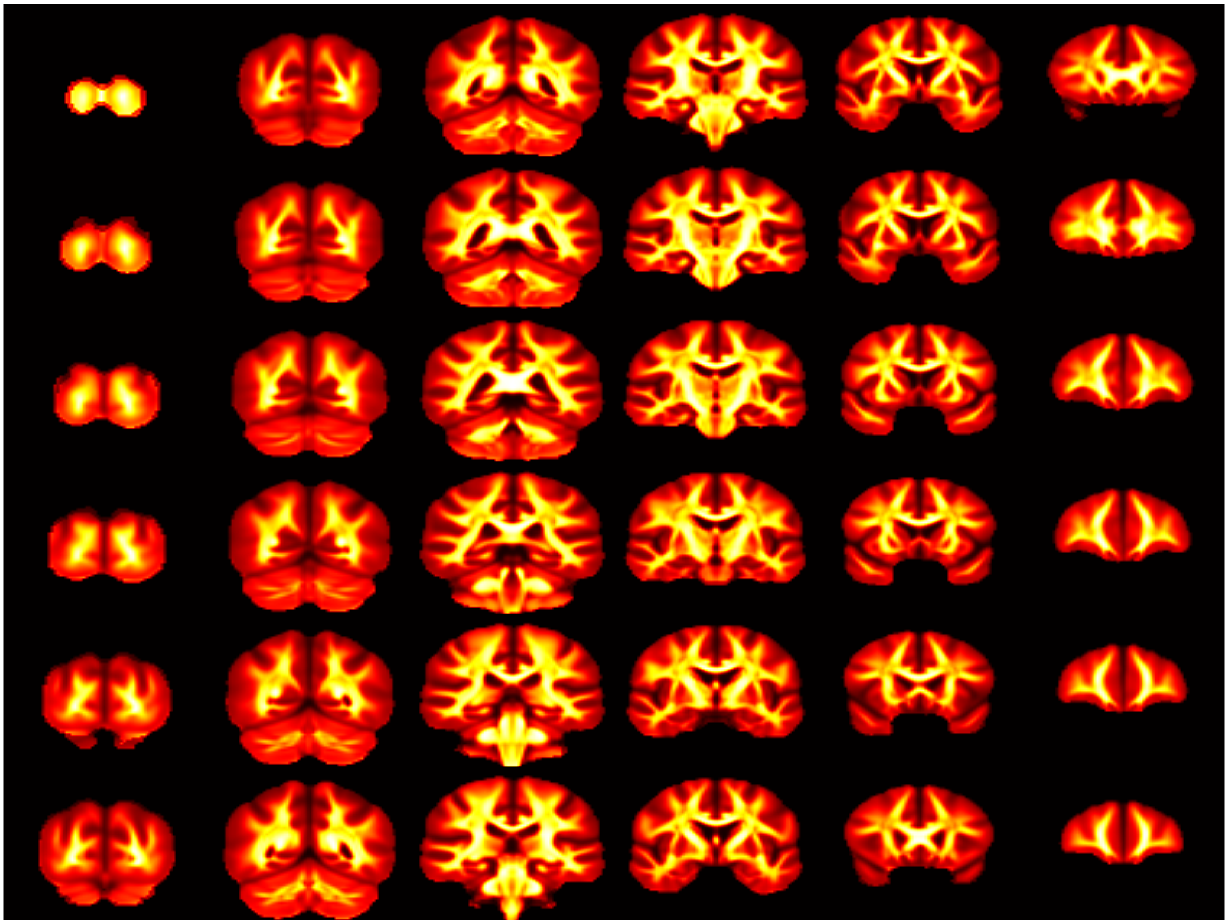

Supplementary Figure 3. Spatial distribution of ND. The ND metrics were averaged across discovery samples of UKB. The coronal sections were rendered slice by slice for every 8 mm. Upper left, first slice from the posterior region. Lower left, last slice from the frontal region.

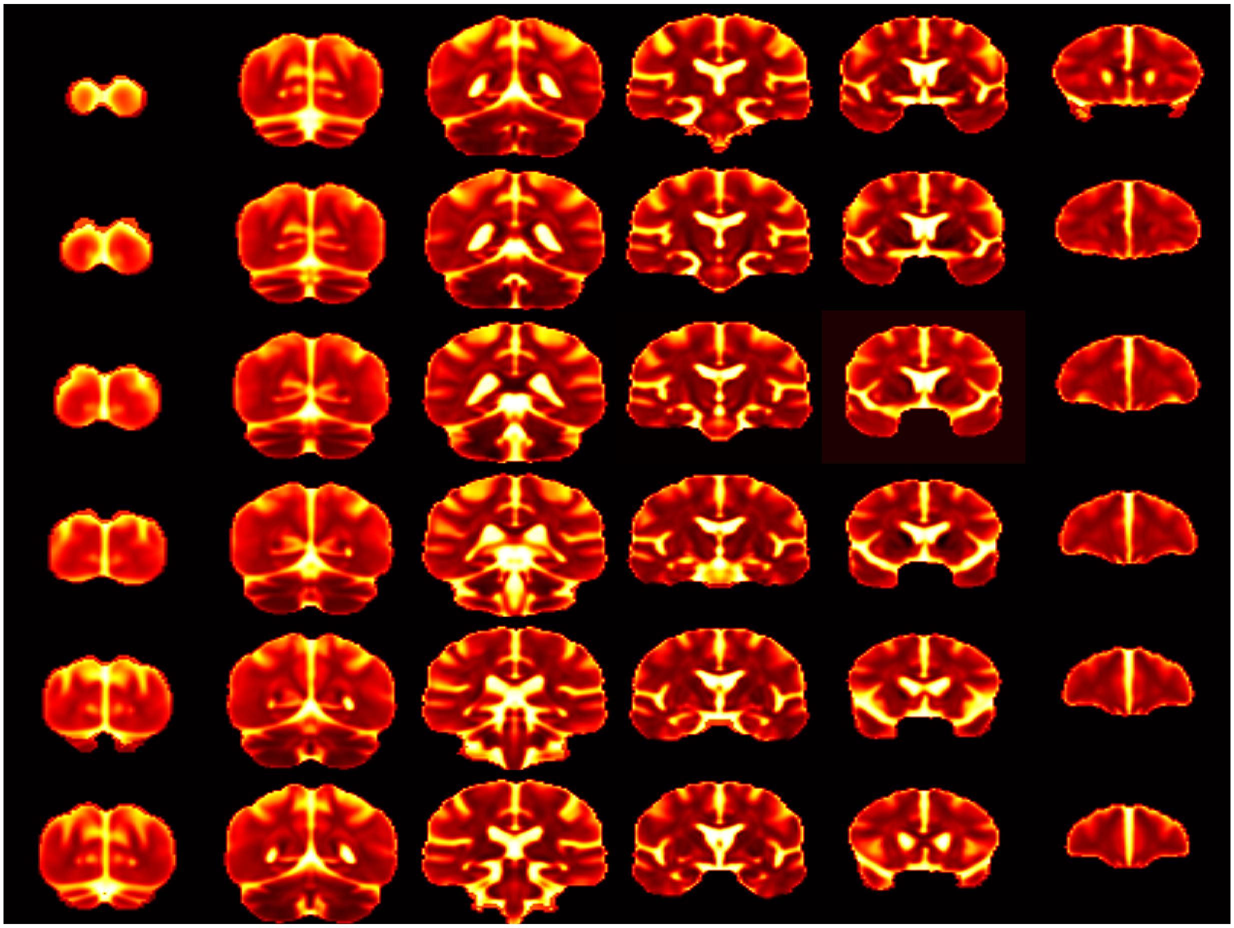

Supplementary Figure 4. Spatial distribution of NF. The NF metrics were averaged across discovery samples of UKB. The coronal sections were rendered slice by slice for every 8 mm. Upper left, first slice from the posterior region. Lower left, last slice from the frontal region.

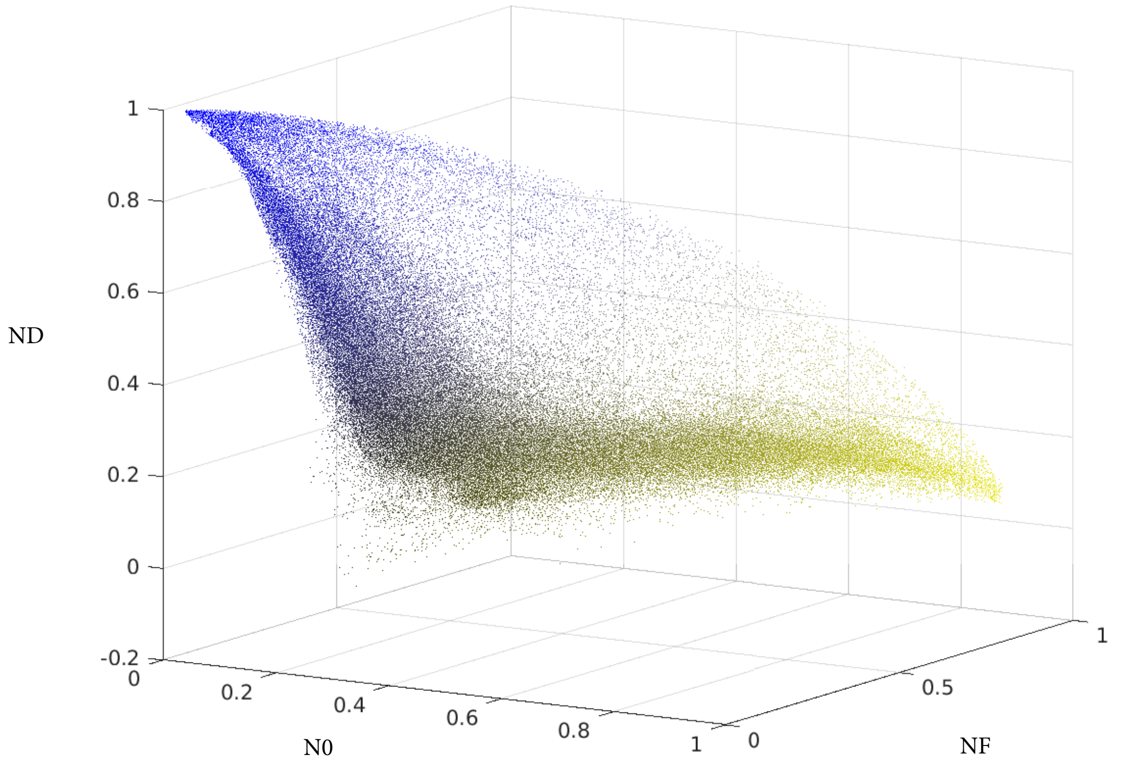

Supplementary Figure 5. Joint distribution of all three tissue sensitive diffusion metrics, N0, ND, and NF.

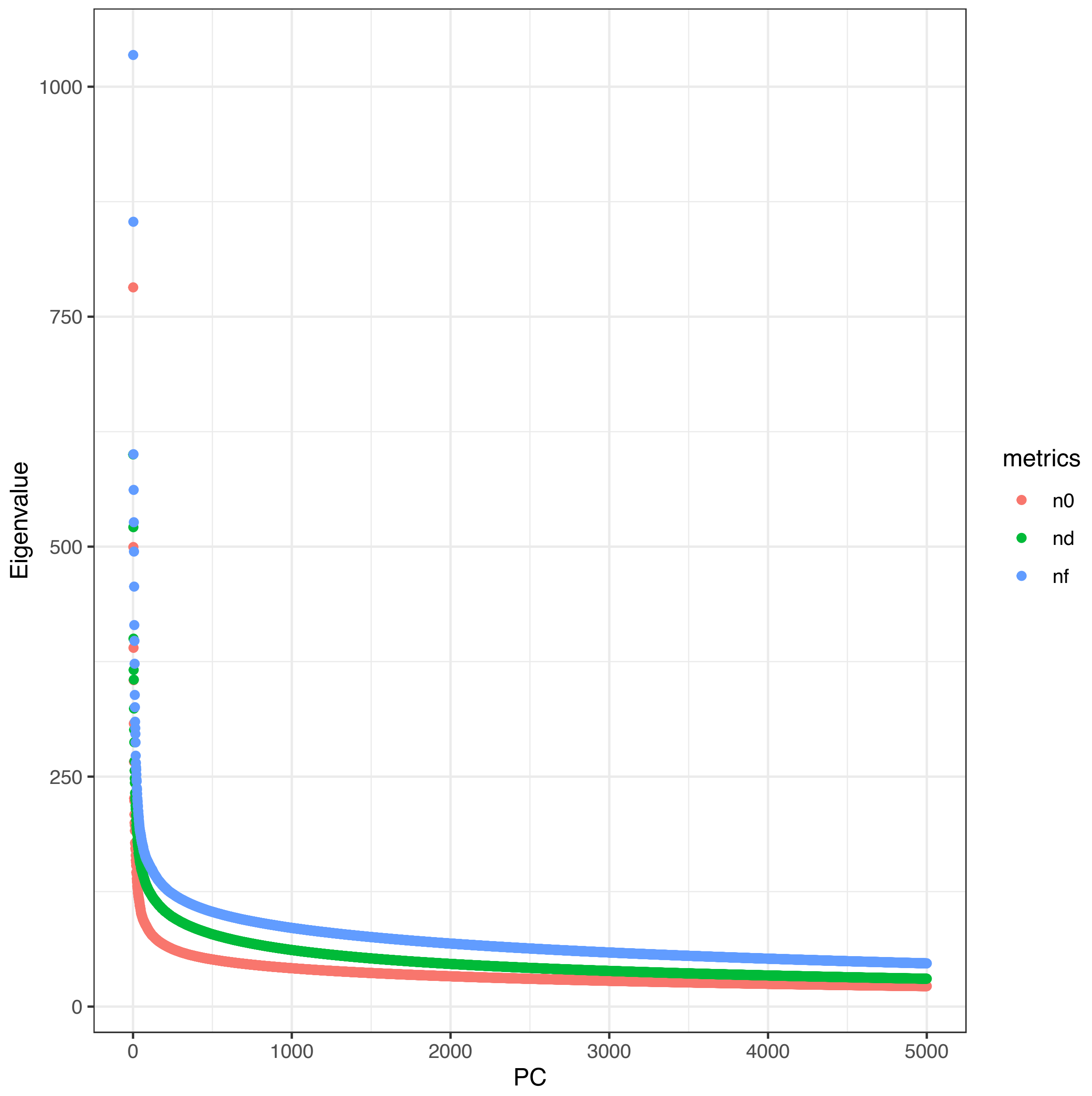

Supplementary Figure 6. Eigenvalues of principal components across three tissue sensitive diffusion metrics

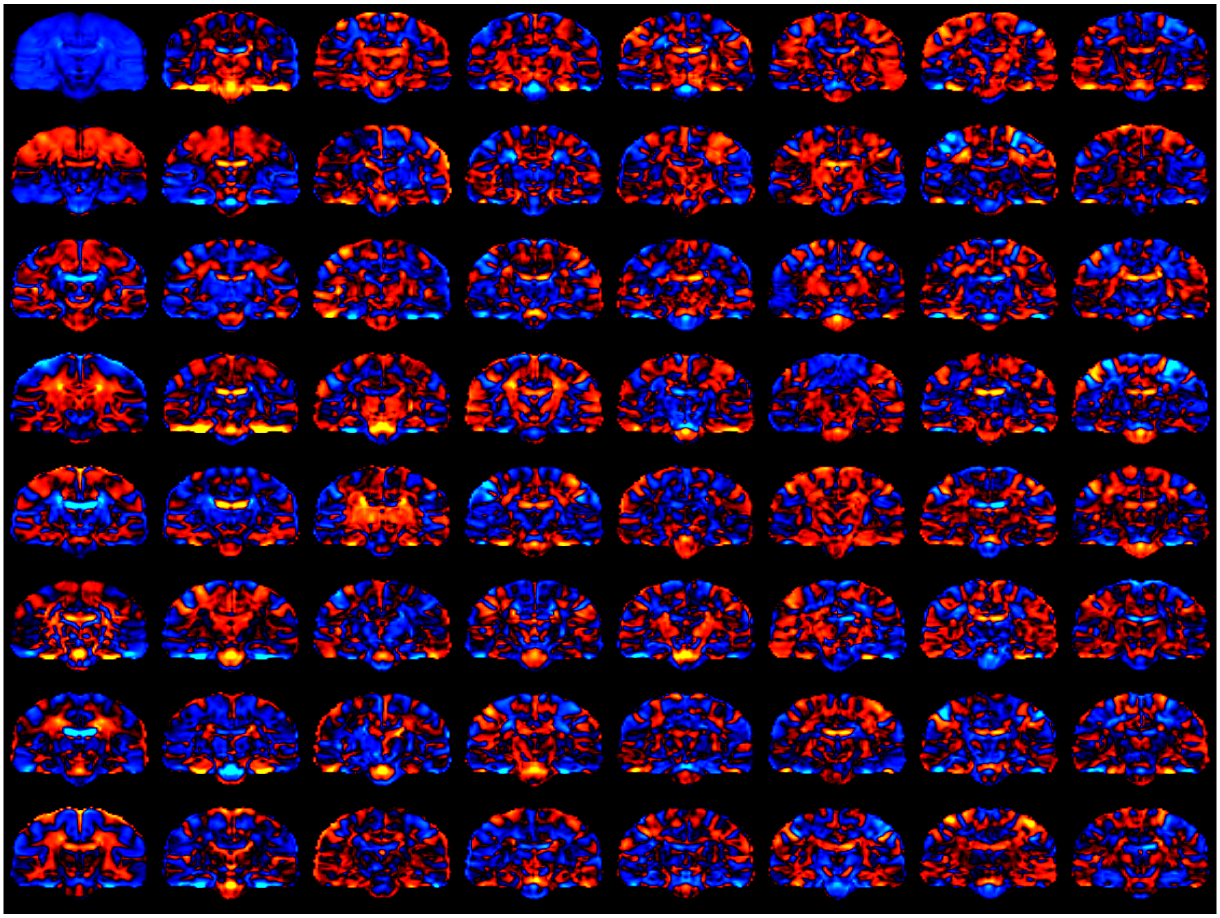

Supplementary Figure 7. Eigenvectors of first 64 principal components of N0. In each frame, the mid-coronal section of the brain was shown, starting from first PC (upper left) to the 64th PC (lower right), ordered accordingly.

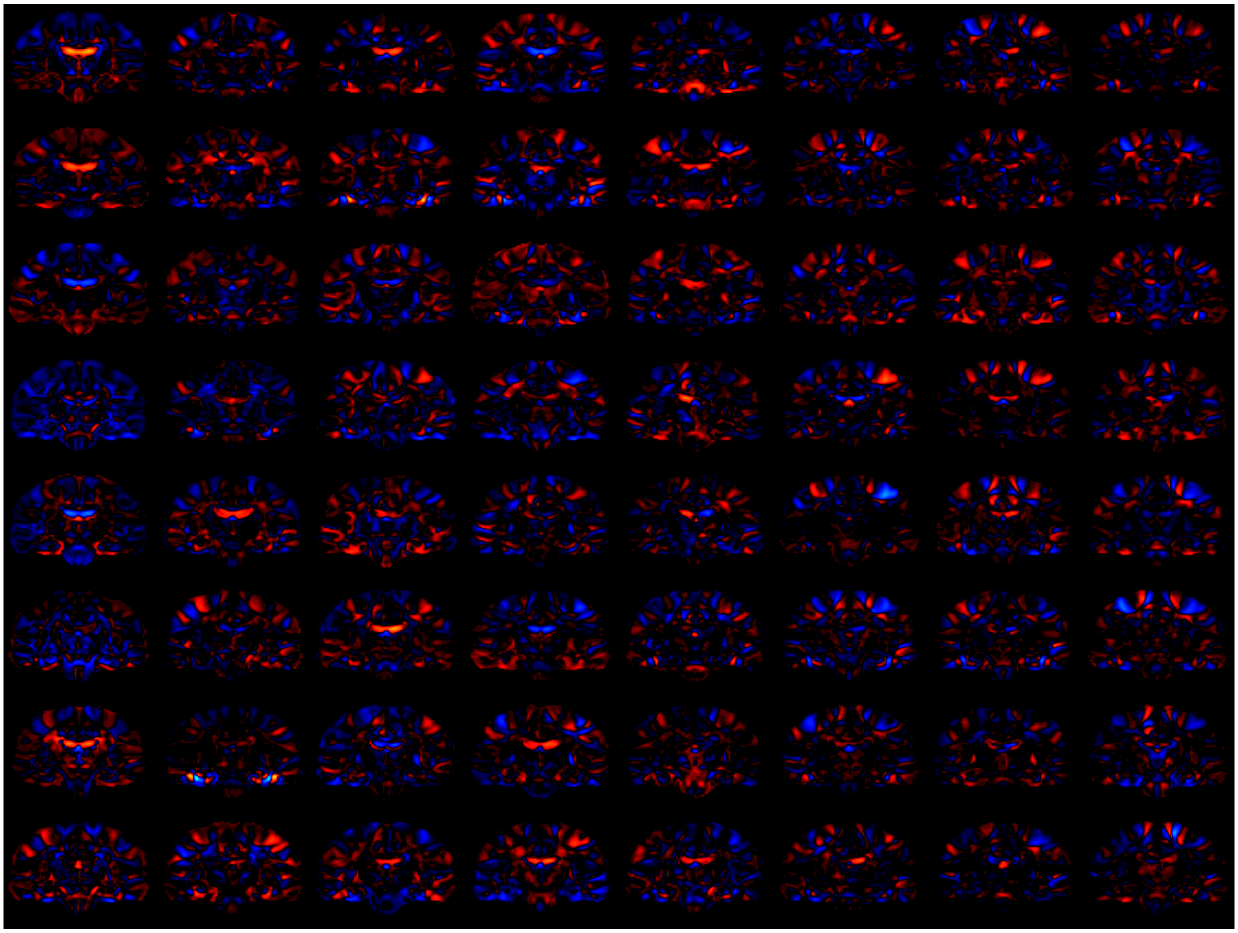

Supplementary Figure 8. Eigenvectors of first 64 principal components of ND. In each frame, the mid-coronal section of the brain was shown, starting from first PC (upper left) to the 64th PC (lower right), ordered accordingly.

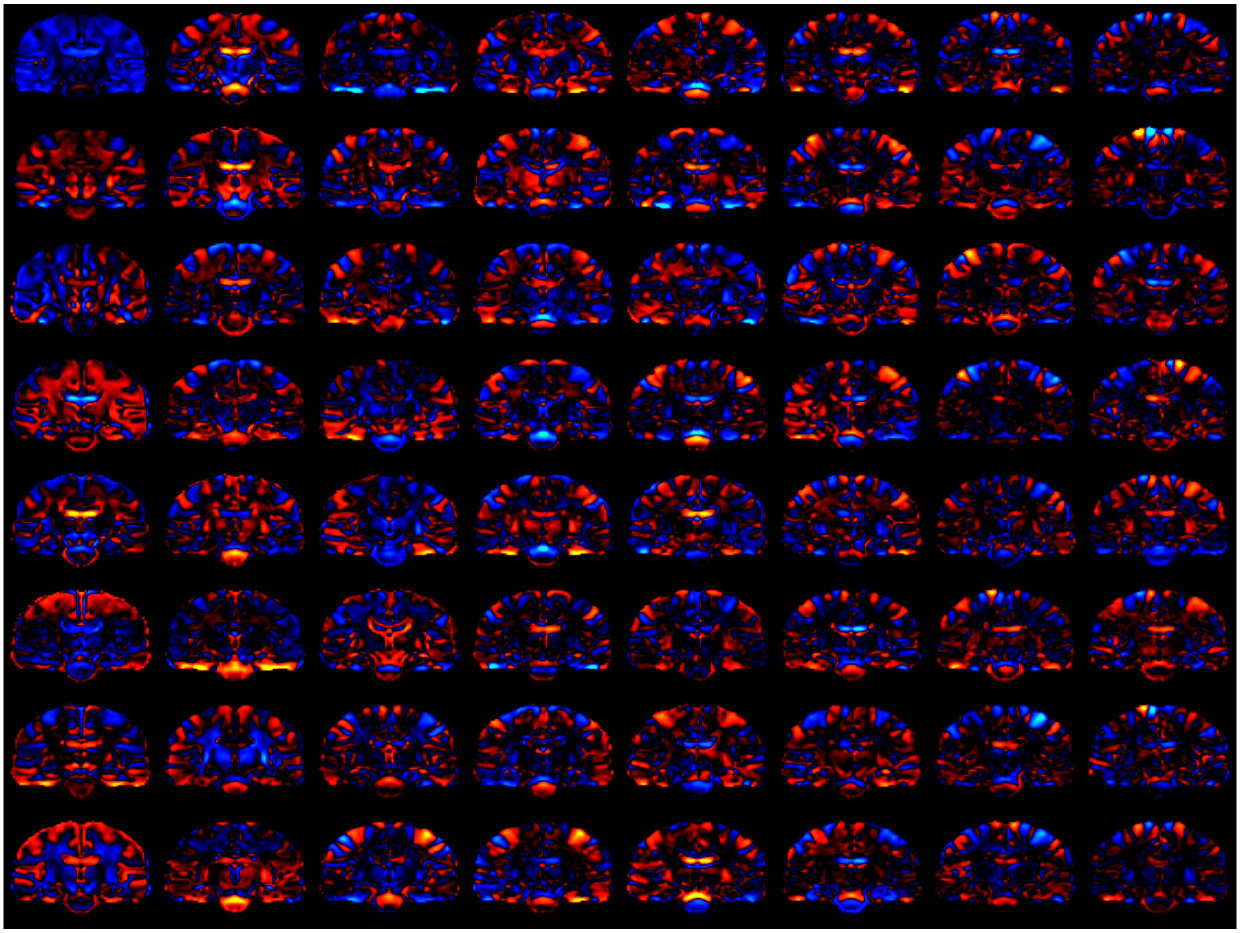

Supplementary Figure 9. Eigenvectors of first 64 principal components of NF. In each frame, the mid-coronal section of the brain was shown, starting from first PC (upper left) to the 64th PC (lower right), ordered accordingly.

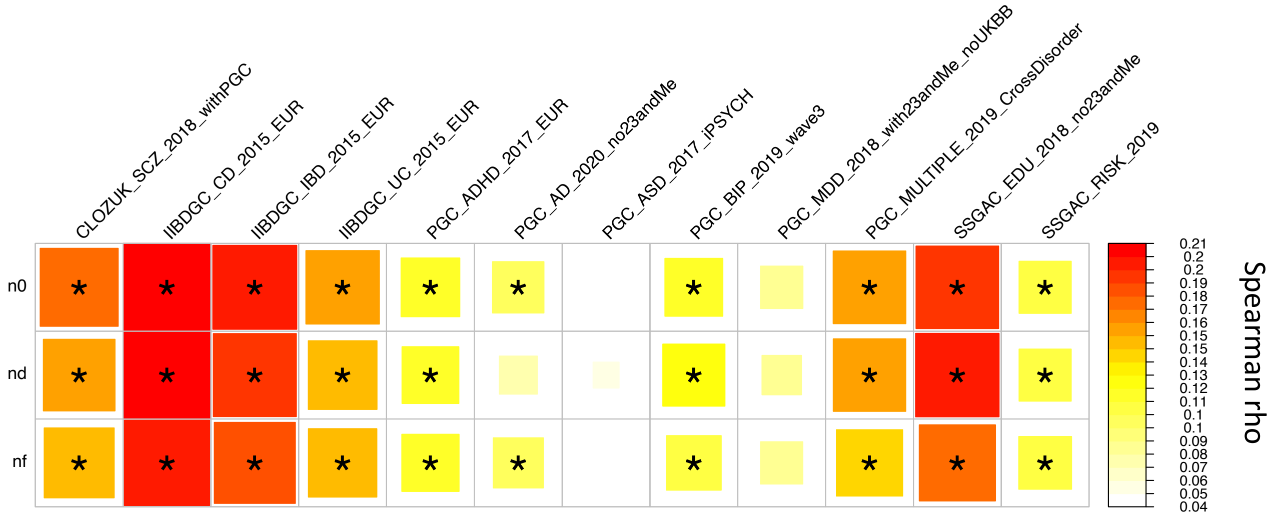

Supplementary Figure 10. Genome-wide signal overlaps between tissue sensitive diffusion metrics and other GWAS, including immune disorders [1], schizophrenia [2], attention deficit hyperactivity disorder [3], bipolar disorder [4], cross-psychiatric-disorders [5], Alzheimer's disease [6], educational attainment [7], and risk-related behaviors [8]

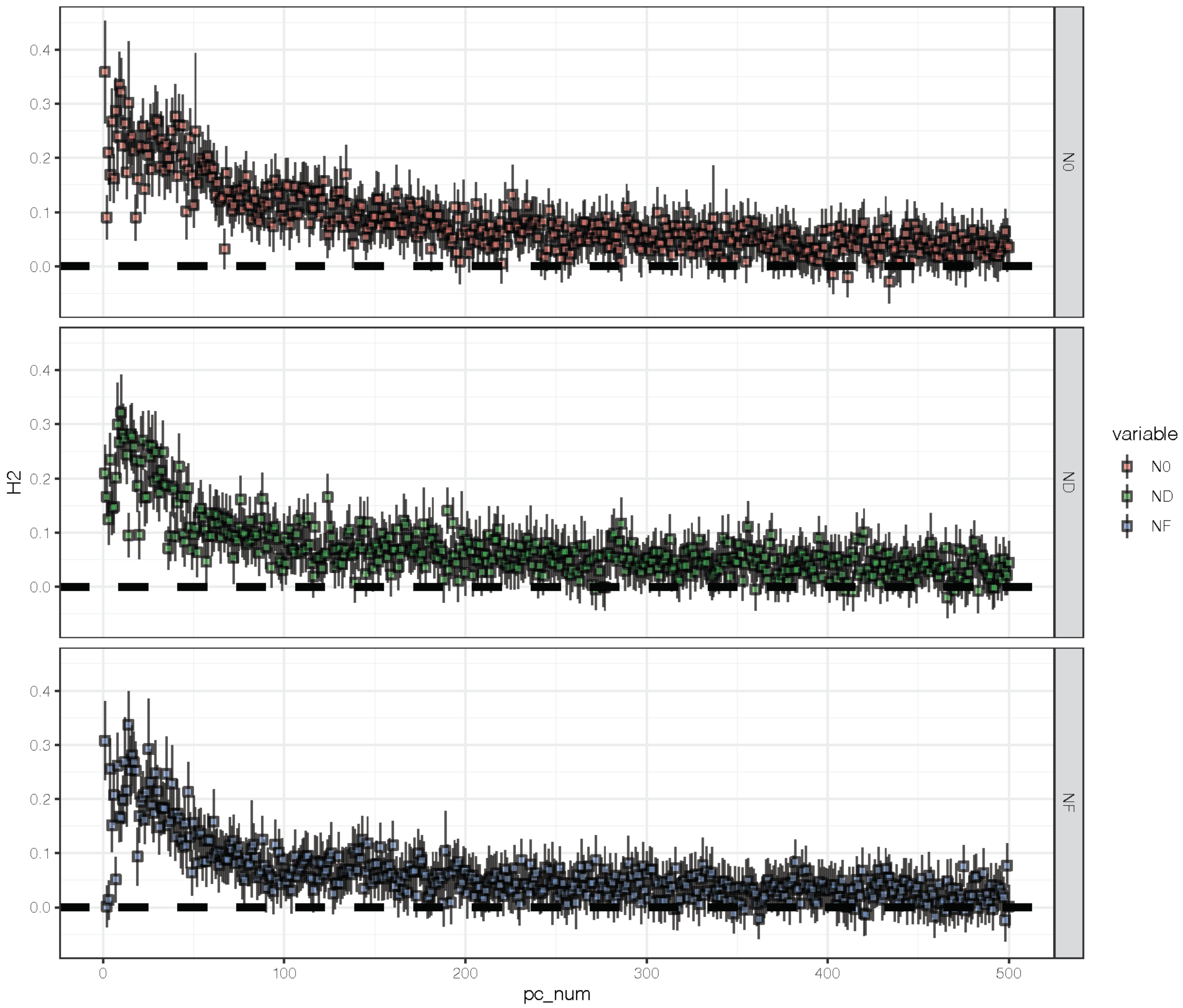

Supplementary Figure 11. SNP-heritability of each principal component across three tissue sensitive diffusion metrics, estimated with LD score regression. LDSC was performed on the summary statistics of each imaging PC, derived from the UKB disocvery GWAS (n = 23,543). The error bars represent 95% confidence interval based on the point estimates and the standard errors from LDSC.

Supplementary References

1 .de Lange, K. M. *et al.* Genome-wide association study implicates immune activation of multiple integrin genes in inflammatory bowel disease. *Nat Genet* **49**, 256-261, doi:10.1038/ng.3760 (2017).

2. Pardiñas, A. F. *et al.* Common schizophrenia alleles are enriched in mutation-intolerant genes and in regions under strong background selection. *Nat Genet* **50**, 381-389, doi:10.1038/s41588-018-0059-2 (2018).

3. Demontis, D. *et al.* Discovery of the first genome-wide significant risk loci for attention deficit/hyperactivity disorder. *Nat Genet* **51**, 63-75, doi:10.1038/s41588-018-0269-7 (2019).

4. Stahl, E. A. *et al.* Genome-wide association study identifies 30 loci associated with bipolar disorder. *Nat Genet* **51**, 793-803, doi:10.1038/s41588-019-0397-8 (2019).

5. Cross-Disorder Group of the Psychiatric Genomics Consortium. Novel Loci and Pleiotropic Mechanisms across Eight Psychiatric Disorders. *Cell* **179**, 1469-1482.e1411, doi:10.1016/j.cell.2019.11.020 (2019).

6. Wightman, D. P. *et al.* Largest GWAS (N=1,126,563) of Alzheimer's Disease Implicates Microglia and Immune Cells. *medRxiv*, 2020.2011.2020.20235275, doi:10.1101/2020.11.20.20235275 (2020).

7. Lee, J. J. *et al.* Gene discovery and polygenic prediction from a genome-wide association study of educational attainment in 1.1 million individuals. *Nat Genet* **50**, 1112-1121, doi:10.1038/s41588-018-0147-3 (2018).

8. Karlsson Linnér, R. *et al.* Genome-wide association analyses of risk tolerance and risky behaviors in over 1 million individuals identify hundreds of loci and shared genetic influences. *Nat Genet* **51**, 245-257, doi:10.1038/s41588-018-0309-3 (2019).
