# Supplementary material for: Multivariate genome-wide association study on tissue-sensitive diffusion metrics highlights pathways that shape the human brain
Source: Nat Commun. 2022 May 3;13:2423. doi: 10.1038/s41467-022-30110-3 (PMC9065144; doi:10.1038/s41467-022-30110-3)
Supplement: Supplementary file 3 — Description of Additional Supplementary Files [file 41467_2022_30110_MOESM3_ESM.pdf]

Description of Additional Supplementary Files

Supplementary Data 1. Unique loci dicovered in current study, indexed based on the genomic physical positions.

Supplementary Data 2. Summary statistics, including mapped genes and overlaps with previous imaging GWAS, of unique loci for N0. P values based on two sided tests obtained from CPC. Loci were defined as 1). reach significant threshold using alpha that controlls family wise error rates across tested phenotypes, and 2). pruned and clumpped based on the criteria specified in the Methods.

Supplementary Data 3. Summary statistics, including mapped genes and overlaps with previous imaging GWAS, of unique loci for ND. P values based on two sided tests obtained from CPC. Loci were defined as 1). reach significant threshold using alpha that controlls family wise error rates across tested phenotypes, and 2). pruned and clumpped based on the criteria specified in the Methods.

Supplementary Data 4. Summary statistics, including mapped genes and overlaps with previous imaging GWAS, of unique loci for NF. P values based on two sided tests obtained from CPC. Loci were defined as 1). reach significant threshold using alpha that controlls family wise error rates across tested phenotypes, and 2). pruned and clumpped based on the criteria specified in the Methods.

Supplementary Data 5. Top five enriched anatomical regions and their corresponding enrichment scores for N0 loci.

Supplementary Data 6. Top five enriched anatomical regions and their corresponding enrichment scores for ND loci.

Supplementary Data 7. Top five enriched anatomical regions and their corresponding enrichment scores for NF loci.

Supplementary Data 8. Region of Interests used in the regional enrichment analysis.

Supplementary Data 9. Druggable gene targets of N0 Locus

Supplementary Data 10. Druggable gene targets of ND Locus

Supplementary Data 11. Druggable gene targets of NF Locus
