# Supplementary material for: Multivariate genome-wide association study on tissue-sensitive diffusion metrics highlights pathways that shape the human brain
Source: Nat Commun. 2022 May 3;13:2423. doi: 10.1038/s41467-022-30110-3 (PMC9065144; doi:10.1038/s41467-022-30110-3)
Supplement: Supplementary file 15 — Reporting Summary [file 41467_2022_30110_MOESM15_ESM.pdf]

nature portfolio

Corresponding author(s): Chun Chieh Fan

Last updated by author(s): Feb 25, 2022

Reporting Summary

Nature Portfolio wishes to improve the reproducibility of the work that we publish. This form provides structure for consistency and transparency in reporting. For further information on Nature Portfolio policies, see our Editorial Policies and the Editorial Policy Checklist.

Statistics

For all statistical analyses, confirm that the following items are present in the figure legend, table legend, main text, or Methods section.

n/a	Confirmed	
☐	☒	The exact sample size (n) for each experimental group/condition, given as a discrete number and unit of measurement
☐	☒	A statement on whether measurements were taken from distinct samples or whether the same sample was measured repeatedly
☐	☒	The statistical test(s) used AND whether they are one- or two-sided<br>*Only common tests should be described solely by name; describe more complex techniques in the Methods section.*
☐	☒	A description of all covariates tested
☐	☒	A description of any assumptions or corrections, such as tests of normality and adjustment for multiple comparisons
☐	☒	A full description of the statistical parameters including central tendency (e.g. means) or other basic estimates (e.g. regression coefficient) AND variation (e.g. standard deviation) or associated estimates of uncertainty (e.g. confidence intervals)
☐	☒	For null hypothesis testing, the test statistic (e.g. F, t, r) with confidence intervals, effect sizes, degrees of freedom and P value noted<br>*Give P values as exact values whenever suitable.*
☒	☐	For Bayesian analysis, information on the choice of priors and Markov chain Monte Carlo settings
☐	☒	For hierarchical and complex designs, identification of the appropriate level for tests and full reporting of outcomes
☐	☒	Estimates of effect sizes (e.g. Cohen's d, Pearson's r), indicating how they were calculated

Our web collection on statistics for biologists contains articles on many of the points above.

Software and code

Policy information about availability of computer code

Data collection	Data is downloaded from the UKB and ABCD data repositories. We used R v3.4 and MATLAB 2017b to process the data into analyzable formats.
Data analysis	ABCD processing codes can be found in github repository series (https://github.com/ABCD-STUDY). Codes used specifically for this study, including obtaining restricted spectrum imaging metrics, combined principal components GWAS, polyvoxel scores, and spatial regional enrichment analyses, can be found in the public accessible GITHUB page at (https://github.com/cmig-research-group/RSIGWAS). The code version used in this study is registered as DOI: 10.5281/zenodo.6289762. The main code base is on MATLAB version 2017b.

For manuscripts utilizing custom algorithms or software that are central to the research but not yet described in published literature, software must be made available to editors and reviewers. We strongly encourage code deposition in a community repository (e.g. GitHub). See the Nature Portfolio guidelines for submitting code & software for further information.

Data

Policy information about availability of data

All manuscripts must include a data availability statement. This statement should provide the following information, where applicable:

- Accession codes, unique identifiers, or web links for publicly available datasets
- A description of any restrictions on data availability
- For clinical datasets or third party data, please ensure that the statement adheres to our policy

Data from UKB is available through UKB application (https://www.ukbiobank.ac.uk) that we obtained under accession number 27412. Adolescent data used in the preparation of this article were obtained from the Adolescent Brain Cognitive Development℠ Study (ABCD Study®) (https://abcdstudy.org), held in the NIMH Data Archive (NDA). ABCD data used in here is under the accession code DOI: 10.15154/1524729. Genomic locus and gene-set results can be found in the Supplementary

Tables.

Field-specific reporting

Please select the one below that is the best fit for your research. If you are not sure, read the appropriate sections before making your selection.

- [x] Life sciences
- [ ] Behavioural & social sciences
- [ ] Ecological, evolutionary & environmental sciences

For a reference copy of the document with all sections, see nature.com/documents/nr-reporting-summary-flat.pdf

Life sciences study design

All studies must disclose on these points even when the disclosure is negative.

Sample size	No statistical methods were used to predetermine the sample size. The samples were selected based on 1). data availability, 2). MRI scan compatibility, 3). successfully passed imaging data quality control, and 4). successfully passed genomic data quality control.
Data exclusions	Basic data quality controls were imposed. Imaging data has to have quality score greater than 0.9 (0 - 1 quality rating). Genomic data has to have call rate greater than 95% and imputation quality score greater than 0.9.
Replication	We designed the study into one discovery set and two independent validation sets. The reproducibility is evaluated based on 1). statistical strength of the associations, 2). significance testing on validation sets given predetermined significance threshold with multiple comparison corrections, and 3). converging biological evidence.
Randomization	No randomness was introduced in sample allocation. Analyses on UKB samples were controlled for age, sex, first 20 genetic principal components, and genotyping batches. Analyses on ABCD samples were controlled for age, sex, first 10 genetic principal components, family relatedness, and ancestral background differences.
Blinding	Investigators were not blinded to the sample allocation. The only groups defined in this analyses is whether individuals belonged to discovery set or validation sets, which researchers in this study needed to know in order to perform analyses.

Reporting for specific materials, systems and methods

We require information from authors about some types of materials, experimental systems and methods used in many studies. Here, indicate whether each material, system or method listed is relevant to your study. If you are not sure if a list item applies to your research, read the appropriate section before selecting a response.

Materials & experimental systems

n/a	Involved in the study	
x		Antibodies
x		Eukaryotic cell lines
x		Palaeontology and archaeology
x		Animals and other organisms
	x	Human research participants
x		Clinical data
x		Dual use research of concern

Methods

n/a	Involved in the study	
x		ChIP-seq
x		Flow cytometry
	x	MRI-based neuroimaging

Human research participants

Policy information about studies involving human research participants

Population characteristics	UKB imaging datasets: 29939 individuals have passed the quality controls. Mean age is 55 and 48.5% of them are Male.<br>ABCD samples: 8189 individuals passed quality controls. Mean age is 9.9 years old and 52% of them are males.
Recruitment	UKB is cohort design, recruiting adults from UK and having MRI scans from three dedicated MRI scanners.<br>ABCD is longitudinal cohort design, recruiting adolescents across 21 sites in USA.
Ethics oversight	NIMH Data Archive and UKBiobank hold the oversight of data usage for this study.

Note that full information on the approval of the study protocol must also be provided in the manuscript.

Magnetic resonance imaging

Experimental design

Design type	No experimental design
Design specifications	NA
Behavioral performance measures	NA

Acquisition

Imaging type(s)	multi-shell diffusion magnetic resonance imaging
Field strength	3
Sequence & imaging parameters	Multi-shell diffusion MRI data of ABCD acquired with seven b=0 s/mm2 frames and 96 non-collinear gradient directions, with 6 directions at b=500 s/mm2, 15 directions at b=1000 s/mm2, 15 directions at b=2000 s/mm2, and 60 directions at b=3000 s/mm2. Multi-shell diffusion MRI data of UKB acquired with five b=0 s/mm2 frames and 100 non-collinear gradient directions, with 50 directions at b=1000 s/mm2 and 50 directions at b=2000 s/mm2.
Area of acquisition	Whole brain scan

Diffusion MRI ☒ Used ☐ Not used

Parameters: Multi-shell diffusion MRI data of ABCD acquired with seven b=0 s/mm2 frames and 96 non-collinear gradient directions, with 6 directions at b=500 s/mm2, 15 directions at b=1000 s/mm2, 15 directions at b=2000 s/mm2, and 60 directions at b=3000 s/mm2. Multi-shell diffusion MRI data of UKB acquired with five b=0 s/mm2 frames and 100 non-collinear gradient directions, with 50 directions at b=1000 s/mm2 and 50 directions at b=2000 s/mm2.

Preprocessing

Preprocessing software	ABCD processing pipeline, implemented in Matlab v.2017a.
Normalization	Intensity normalization, rigid body registration, and none-linear multi-channel registrations were performed to ensure the compatibility in the voxel level.
Normalization template	The common atlas used for registration is in RAS, while the study specific group average were obtained iteratively.
Noise and artifact removal	Processed through forward-reverse gradient warping, eddy current correction, and motion correction to reduce the spatial distortion and signal heterogeneities driven by scanner differences.
Volume censoring	No censoring

Statistical modeling & inference

Model type and settings	Multivariate genomewide assoications were performed. The discovery inference is based on combined principal components associations, which is a weighted-sum test. The validation is based on the polyvoxel scoring, which is the sum of regression coefficients across voxels from the discovery set. Fixed effects multiple regressions were performed in UKB set while the mixed effects models were used in analyzing ABCD data.
Effect(s) tested	NA

Specify type of analysis: ☒ Whole brain ☐ ROI-based ☐ Both

Statistic type for inference (See Eklund et al. 2016)	Weighted sum test with combined principal components analyses.
Correction	FWE

Models & analysis

n/a	Involved in the study
☒	☐ Functional and/or effective connectivity
☒	☐ Graph analysis
☐	☒ Multivariate modeling or predictive analysis

Multivariate modeling and predictive analysis	Imaging voxels were treated as multivariate dependent variables while the independent variables include genetic variants and study specific covariates.
